# Maternal Anemia as a Predictor of Anemia in the Child in Nepal: An Analysis of the Demographic and Health Survey

**DOI:** 10.7759/cureus.89229

**Published:** 2025-08-01

**Authors:** Yasutaka Kuniyoshi

**Affiliations:** 1 Department of Social Services and Healthcare Management, International University of Health and Welfare, Otawara, JPN

**Keywords:** anemia, anemia in the child, hemoglobin level, machine learning, maternal anemia, nepal demographic health survey, shap

## Abstract

This study aims to evaluate the extent to which maternal anemia predicts anemia in the child. This secondary data analysis used the Nepal Demographic and Health Survey datasets from 2011, 2016, and 2022. The study included children aged six to 59 months of age and their mothers from households eligible for blood testing. We performed multiple logistic regression analyses to determine adjusted odds ratios (ORs) for the association between maternal anemia and anemia in the child. We used the child's age, gender, birth as a twin, birth order, and body mass index (BMI), as well as maternal age, BMI, education level, wealth index, and place of residence as variables for adjustment covariates. We used eXtreme Gradient Boosting (XGBoost) to develop the prediction model for anemia in the child. We utilized the SHapley Additive exPlanations (SHAP) method to explain the importance of predictors. Children with moderate-to-severe anemia (hemoglobin level <10.0 g/dL) accounted for 21% of the studied population. Maternal anemia demonstrated a positive association with anemia in the child (adjusted OR: 2.26; 95% confidence interval: 1.84-2.76). It demonstrated the second most substantial effect on the prediction model, succeeding the child's age, within the SHAP framework for the XGBoost model. Higher maternal hemoglobin levels (g/dL) were associated with a lower likelihood of anemia in the child, shifting the prediction toward nonanemic status. Maternal anemia is thus a significant predictor of anemia in the child. Determining the presence of anemia in the child may be important in instances where the mother is diagnosed with anemia. Comprehensive interventions targeting mothers and the entire household may be indispensable in ameliorating anemia in children from a public health perspective.

## Introduction

Anemia is a public health problem in many low- and middle-income countries (LMICs). It affects 30% of women and 40% of children under five years of age globally [1,2]. Iron deficiency anemia is the leading cause of childhood anemia in these countries, while other nutritional deficiencies, including folic acid and vitamin B12, chronic infections, malaria, and blood disorders, such as sickle cell disease, contribute to the etiology of anemia. Iron deficiency anemia may adversely affect cognitive and physical development, school performance, and immune function in children, with significant economic consequences [3]. Substantial evidence indicates that anemia demonstrates a significant negative effect on a country’s socioeconomic productivity by reducing learning and work capacity [4,5].

Childhood anemia is a serious nutritional problem, especially in LMICs, particularly in Africa and Southeast Asia [2]. In 2021, the World Health Organization (WHO) reported that the prevalence of childhood anemia in Nepal was average for South East Asia, with an incidence rate of 46.1% [2]. Several interventions, including expanded availability of multimicronutrient powders, biannual deworming of children aged one to five years with vitamin A supplementation, and promotion of insecticide-treated bed nets for children under five living in malaria-endemic areas, were implemented to reduce the incidence of childhood anemia [6,7]. However, the proportion of children suffering from moderate-to-severe anemia remains high, fluctuating between 19% and 27%, despite efforts by the Nepal Government [8]. Therefore, identifying children at increased risk of moderate-to-severe anemia who require early intervention and treatment is important, in addition to continuing preventive strategies.

Several studies conducted in Nepal revealed a positive association between maternal anemia and the incidence of anemia in the child [9-11]. However, the intricacies of this association in the context of other variables remain unknown. Therefore, this study aimed to investigate the association between maternal anemia and anemia in the child while controlling for other covariates, and to quantify the predictive importance of maternal hemoglobin level using a machine learning technique. Furthermore, we sought to explore how these findings could inform the development of more targeted public health interventions at the household and community levels. By elucidating this link, this study aims to provide actionable evidence for strategies that address maternal and child health in a more integrated manner.

## Materials and methods

Data source and participants

This secondary data analysis used Nepal Demographic and Health Survey (DHS) datasets from 2011 [6], 2016 [7], and 2022 [8]. The DHS is a cross-sectional survey conducted by the Ministry of Health and Population/Nepal, Nepal Economic, Agricultural, and Research Extension (New ERA/Nepal), and ICF International. The study included children aged six to 59 months and their mothers from households that were eligible for blood testing. The study excluded children with pregnant mothers or those with missing information on maternal hemoglobin levels. This exclusion was implemented because physiological hemodilution during pregnancy temporarily lowers maternal hemoglobin levels, which could act as a confounder in the analysis of the association between maternal anemia and anemia in the child. We conducted a complete-case analysis, and individuals with missing data on any of the covariates included in the models were also excluded from the final analysis. No imputation methods were used. The Nepal DHS program was approved by the Ethics Committee of the Nepal Health Research Council. The DHS program obtained written informed consent from each individual before interviewing participants. The DHS data set is publicly available, and we requested and obtained it from ICF's DHS program website for secondary analysis.

Anemia

Assessment of anemia was based on hemoglobin levels. A portable battery-operated device (HemoCue® system, Danaher Corporation, Sweden) was used to analyze the hemoglobin levels in children on-site. Blood samples were collected via capillary puncture from the finger or from the finger or heel in participants aged six to 11 months.

Anemia was defined as a hemoglobin level of <10 g/dL, equivalent to moderate-to-severe anemia, as per the WHO definition. This is despite the WHO defining anemia in children aged six to 59 months as hemoglobin <11 g/dL (mild anemia, according to the WHO definition) [12]. The definition of <10 g/dL was selected to identify children with anemia who should be investigated for anemia etiology or treated early. This specific cutoff was chosen to focus the analysis on children with moderate-to-severe anemia, as they represent a high-priority group requiring immediate clinical investigation and treatment. While this approach excludes cases of mild anemia (hemoglobin 10.0-10.9 g/dL), it allows for the identification of predictors for more clinically significant conditions. It is important to note that this definition may result in a lower prevalence estimate compared to studies using the standard WHO threshold of <11 g/dL.

Covariates

Based on previous studies [9-11],we selected the following variables as covariates: age, child's gender, birth order, birth as a twin, and body mass index (BMI), and maternal age, BMI, education level, wealth index, and place of residence.

Statistical Analysis

R software (version 4.3.2, R Foundation for Statistical Computing, Vienna, Austria, https://www.R-project.org/) and Python (version 3.10.12, Python Software Foundation, Fredericksburg, VA, US) were used for all the statistical analyses. We compared children with and without anemia based on anthropometric and sociodemographic characteristics. We performed multiple logistic regression analyses to calculate the adjusted odds ratios (aORs) for the association between maternal anemia and anemia in the child. We used the following variables as adjustment factors: child's age (categorized as 6-11, 12-23, 24-35, 36-47, or 48-59 months), child's gender (male or female), twin (yes or no), birth order (first or not), child's BMI (categorized as severe underweight, moderate underweight, normal, or overweight), maternal age (categorized as 15-19, 20-24, 25-29, 30-34, 35-39, 40-44, or ≥45 years), maternal BMI (categorized as moderate to severe thin, mild thin to normal, or overweight), maternal education level (categorized as no education, basic, secondary, or higher), wealth index (categorized as poorest, poorer, middle, richer, or richest), and place of residence (rural or urban). Additionally, we conducted subgroup analyses, stratified by age.

We used eXtreme Gradient Boosting (XGBoost) to develop a prediction model for anemia in the child. XGBoost is a scalable tree-boosting system known for its strong generalizability and high prediction performance [13]. We used the child's gender (male or female), twin (yes or no), birth order (first or not), maternal education level (categorized as no education, basic, secondary, or higher), wealth index (categorized as poorest, poorer, middle, richer, or richest), and place of residence (rural or urban), including continuous variables, such as maternal hemoglobin level (g/dL), child's age (months), child's BMI (standard deviation), maternal age (years), and maternal BMI (kg/m^2^) as predictors.

The performance of the model was assessed using spatial validation by splitting the survey data. Specifically, we trained the model on two survey datasets and used the remaining one as a test dataset to obtain the evaluation index. This process was repeated thrice, with the results averaged. Scikit-learn’s [14] GridSearchCV function was used to identify the best hyperparameter combination. We utilized the SHapley Additive explanation (SHAP) framework to interpret the prediction model [15]. Given our use of a tree-based model, we applied the "TreeExplainer function" from the SHAP Python package. To visualize the global feature importance and the direction of feature impacts, SHAP summary plots were generated using the package's default settings. Additionally, subgroup analyses were conducted, stratified by child's age.

## Results

**Figure 1:**
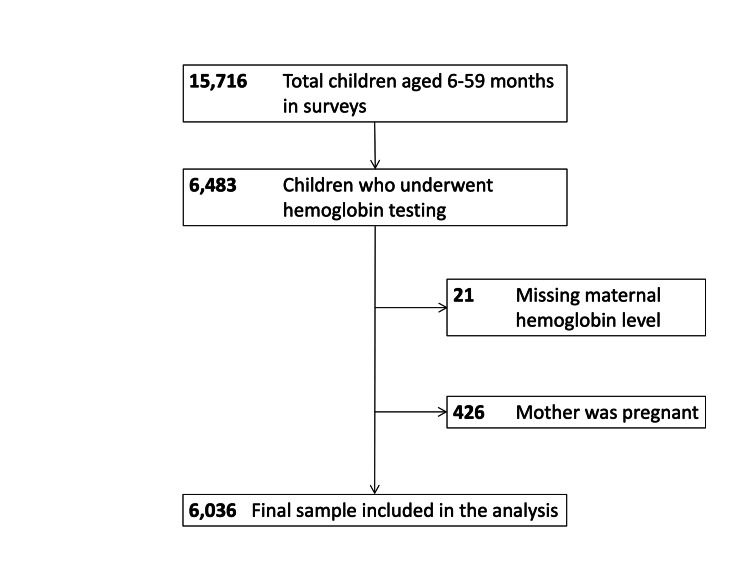
Flowchart of participant selection A flowchart detailing the selection process for the final study population from the Nepal Demographic and Health Survey datasets. Children aged 6-59 months were excluded if they did not undergo hemoglobin testing, or if their mother was pregnant or had missing hemoglobin data.

Among the 15,716 children aged six to 59 months, the analysis included a subset of 6,036 children and their mothers (Figure 1). 

The prevalence of anemia in children, defined as a hemoglobin level of <10.0 g/dL, was 21% (1,291/6,036). Table 1 shows the distribution and proportion of children with anemia across various covariates.

**Table 1:** Characteristics of children aged six to 59 months and their mothers, stratified by the child's anemia status *includes mild anemia with hemoglobin levels of 10.0–11.0 g/dL; OR: odds ratio; CI: confidence interval; Hb: hemoglobin; BMI: body mass index We used multivariable logistic regression analysis to calculate adjusted ORs, controlling for the following covariates: child's age (6–11, 12–23, 24–35, 36–47, 48–59), child's gender (male or female), twin (yes or no), birth order (first or not), child's BMI (severe underweight, moderate underweight, normal, or overweight), and maternal age (15–19, 20–24, 25–29, 30–34, 35–39, 40–44, or ≥45 years of age), maternal BMI (moderate to severe thin, mild thin to normal, or overweight), maternal education level (no education, basic, secondary, or higher), wealth index (poorest, poorer, middle, richer, richest), and place of residence (rural or urban).

	Not anemia* (Hb≥10.0 g/dL)	Anemia (Hb<10.0 g/dL)	Adjusted OR (95% CI)
Survey year			
2011	1,543/1,931 (80%)	388/1,931 (20%)	
2016	1,434/1,946 (74%)	512/1,946 (26%)	
2022	1,768/2,159 (82%)	391/2,159 (18%)	
Gender			
Male	2,505/3,181 (79%)	676/3,181 (21%)	Reference
Female	2,240/2,855 (78%)	615/2,855 (22%)	0.99 (0.87–1.13)
Child's age (months)			
6–11	397/662 (60%)	265/662 (40%)	7.82 (6.04–10.2)
12–23	886/1,345 (66%)	459/1,345 (34%)	5.72 (4.55–7.23)
24–35	1,103/1,372 (80%)	269/1,372 (20%)	2.64 (2.08–3.36)
36–47	1,190/1,372 (87%)	182/1,372 (13%)	1.63 (1.27–2.10)
48–59	1,169/1,285 (91%)	116/1,285 (9%)	Reference
Child's BMI			
Severe underweight	58/78 (74%)	20/78 (26%)	0.74 (0.42–1.26)
Moderate underweight	235/328 (72%)	93/328 (28%)	1.06 (0.81–1.38)
Normal	4,381/5,542 (79%)	1,161/5,542 (21%)	Reference
Overweight	47/56 (84%)	9/56 (16%)	0.71 (0.32–1.42)
Twin			
Yes	44/66 (67%)	22/66 (33%)	2.05 (1.16–3.54)
No	4,701/5,970 (79%)	1,269/5,970 (21%)	Reference
Birth order			
First	1,520/1,909 (80%)	389/1,909 (20%)	Reference
≥Second	3,225/4,127 (78%)	902/4,127 (22%)	0.99 (0.84–1.18)
Maternal age (years)			
15–19	229/334 (69%)	105/334 (31%)	1.10 (0.83–1.44)
20–24	1,557/1,993 (78%)	436/1,993 (22%)	Reference
25–29	1,627/2,046 (80%)	419/2,046 (20%)	1.13 (0.95–1.34)
30–34	820/1,019 (80%)	199/1,019 (20%)	1.07 (0.86–1.32)
35–39	335/430 (78%)	95/430 (22%)	1.19 (0.89–1.59)
40–44	143/170 (84%)	27/170 (16%)	0.89 (0.55–1.39)
45–	34/44 (77%)	10/44 (23%)	1.40 (0.61–2.93)
Maternal BMI			
Moderate to Severe thin	222/319 (70%)	97/319 (30%)	1.43 (1.09–1.86)
Normal (Mild thin to Norma)	3,782/4,822 (78%)	1,040/4,822 (22%)	Reference
Overweight	739/891 (83%)	152/891 (17%)	0.89 (0.72–1.09)
Maternal education level			
No education	1,556/2,049 (76%)	493/2,049 (24%)	1.32 (1.11–1.58)
Basic	1,205/1,511 (80%)	306/1,512 (20%)	Reference
Secondary	1,598/2,013 (79%)	415/2,013 (21%)	0.98 (0.82–1.18)
Higher	616/732 (84%)	116/732 (16%)	0.80 (0.58–1.08)
Wealth index			
Poorest	1,446/1,817 (80%)	371/1,817 (20%)	0.85 (0.70–1.03)
Poorer	1,003/1,287 (78%)	284/1,287 (22%)	0.92 (0.76–1.13)
Middle	911/1,189 (77%)	278/1,189 (23%)	Reference
Richer	769/1,011 (76%)	242/1,011 (24%)	1.04 (0.84–1.29)
Richest	617/733 (84%)	116/733 (16%)	0.74 (0.56–0.97)
Household size			
2–4	1,521/1,872 (81%)	351/1,872 (19%)	Reference
5–7	2,232/2,840 (79%)	608/2,840 (21%)	1.09 (0.93–1.28)
≥8	992/1,324 (75%)	332/1,324 (25%)	1.24 (1.03–1.49)
Residence			
Urban	2,009/2,551 (79%)	542/2,551 (21%)	Reference
Rural	2,736/3,485 (79%)	749/3,485 (21%)	0.93 (0.81–1.07)
Maternal anemia			
Not anemia	4,386/5,483 (80%)	1,097/5,483 (20%)	Reference
Anemia	359/553 (65%)	194/553 (35%)	2.26 (1.84–2.76)

The prevalence of anemia among children by age group was higher in younger children and varied widely from 9% to 40%. Maternal anemia was positively linked with the child's anemia (adjusted OR: 2.26; 95% confidence interval: 1.84-2.76). This association persisted across different age groups (Table 2).

**Table 2:** Association between maternal anemia and anemia in the child stratified by the child's age CI: confidence interval ^a^Adjusted for child's gender (male or female), twin (yes or no), birth order (first or not), child's BMI (severe underweight, moderate underweight, normal, or overweight), maternal age (15–19, 20–24, 25–29, 30–34, 35–39, 40–44, or ≥45 years of age), maternal BMI (moderate to severe thin, mild thin to normal, or overweight), maternal education level (no education, basic, secondary, or higher), wealth index (poorest, poorer, middle, richer, richest), and place of residence (rural or urban)

Child's age (months)	Adjusted odds ratio (95% CI)	Crude risk ratio
Total (6–59)	2.26 (1.84–2.76)	1.75
6–11	2.08 (1.16–3.81)^a^	1.47
12–23	1.98 (1.35–2.91)^a^	1.57
24–35	2.47 (1.62–3.72)^a^	2.00
36–47	2.40 (1.49–3.78)^a^	2.29
48–59	2.63 (1.56–4.30)^a^	2.38

**Figure 2:**
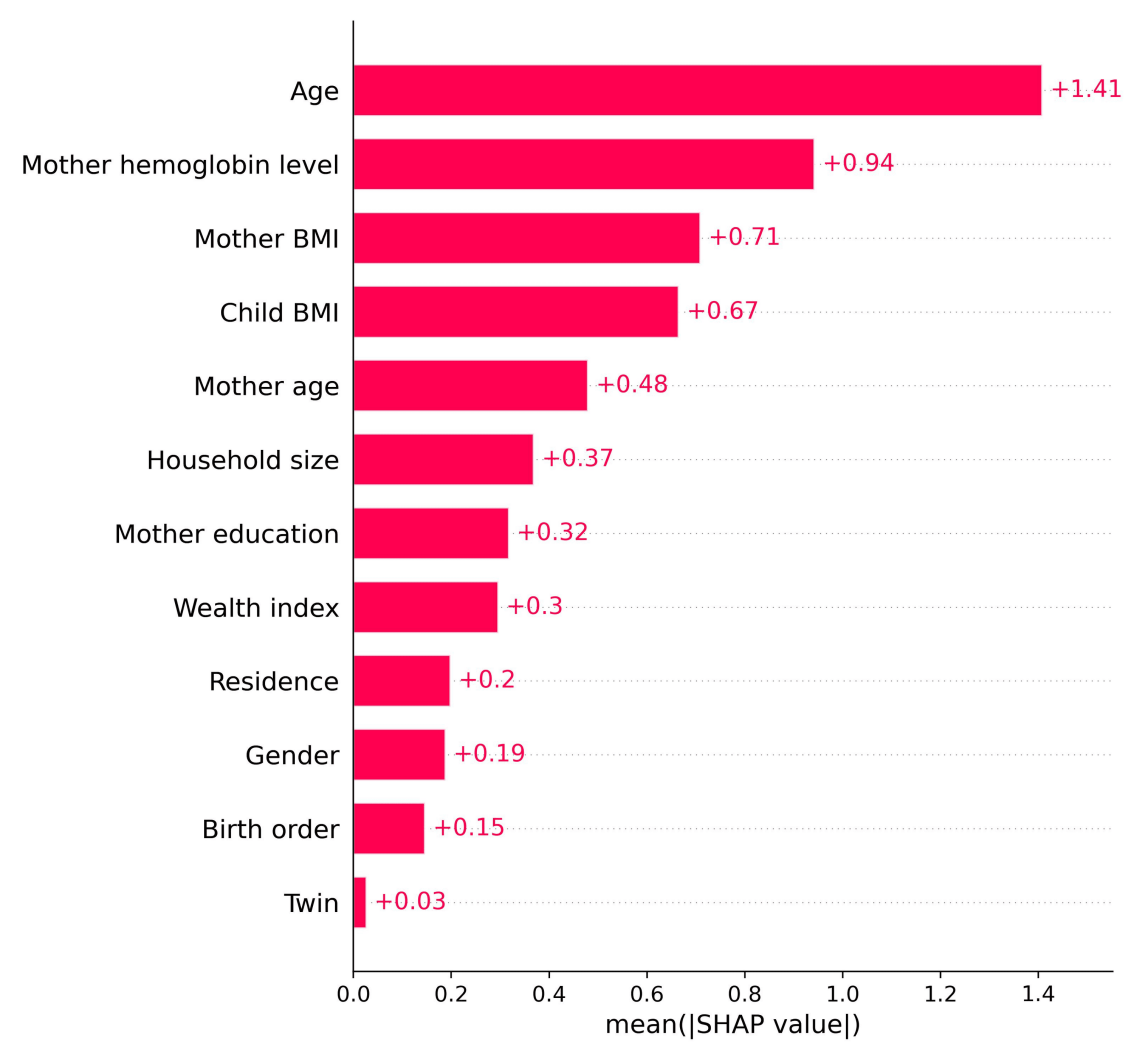
Contribution of variables identified by XGBoost and SHAP: The importance ranking of the variables based on the mean (|SHAP value|) XGBoost: eXtreme Gradient Boosting; SHAP: SHapley Additive exPlanations

The prediction models, developed using the basic XGB, demonstrated a mean area under the receiver operating characteristic curve, F1 score, sensitivity, and specificity of 0.56, 0.28, 24%, and 89%, respectively. The variable importance plot (Figure 2) ranks the variables based on their contribution to the prediction model in descending order.

Notably, maternal hemoglobin level appeared as the second strongest predictor, after the child’s age.

**Figure 3:**
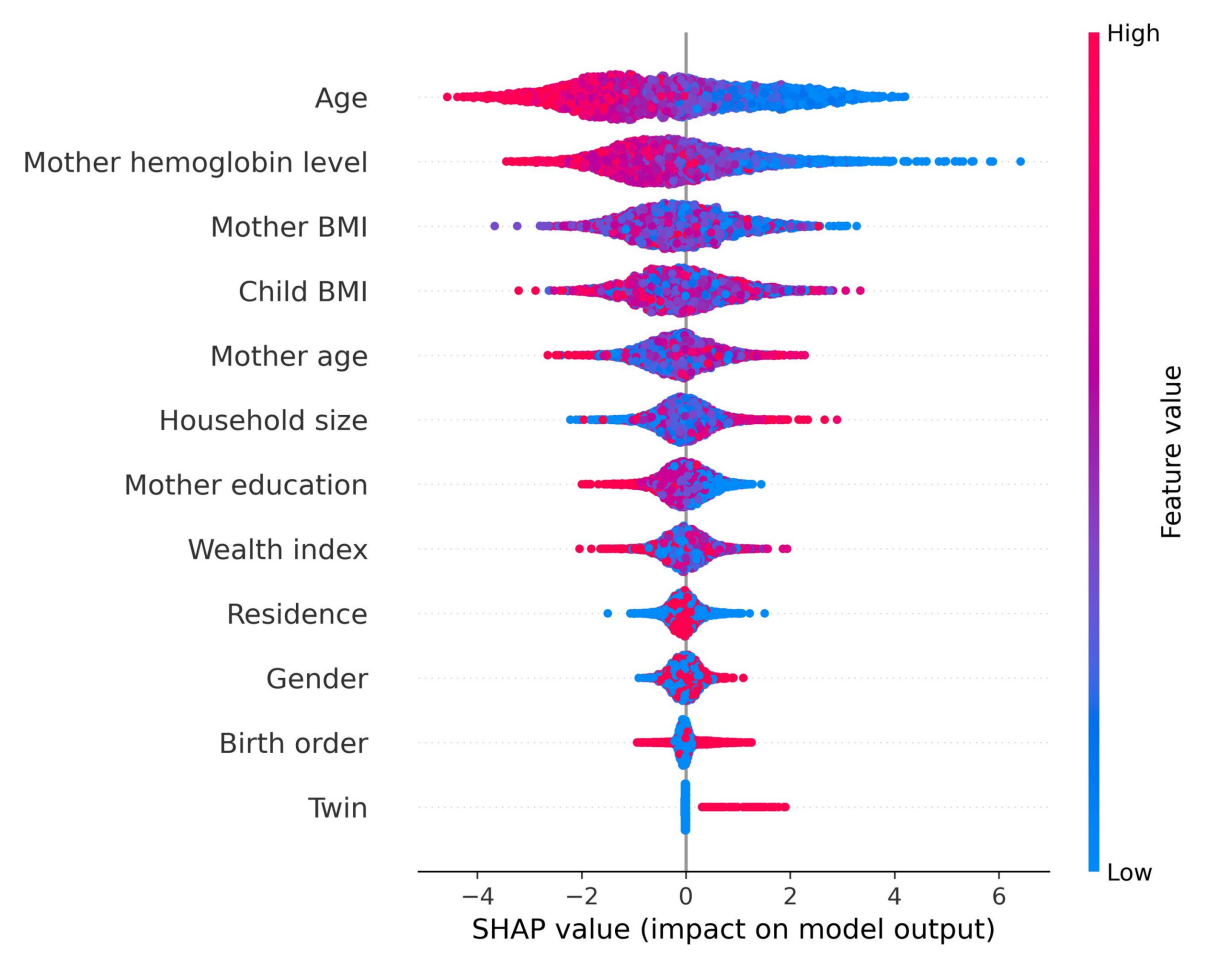
Contribution of variables identified by XGBoost and SHAP: SHAP beeswarm summary plot on the effect of variable XGBoost: eXtreme Gradient Boosting; SHAP: SHapley Additive exPlanations Dots indicate distinct patient cases, color-coded according to the value of the corresponding variable on the Y-axis and their associated Shapley value on the X-axis. Both the magnitude and directionality of contribution to the prediction of anemia in the child are indicated while maintaining the ranking order of importance.

The SHAP beeswarm summary plot shows that an increase in both the child’s age (in months) and the maternal hemoglobin level (in g/dL) negatively affects the prediction, shifting it toward nonanemic outcomes (Figure 3).

The horizontal position of a point in this plot indicates the association of the effect of the value with the level of the predicted outcome. The color coding, with red indicating higher values and blue representing lower values, provides additional insights into each variable.

**Figure 4:**
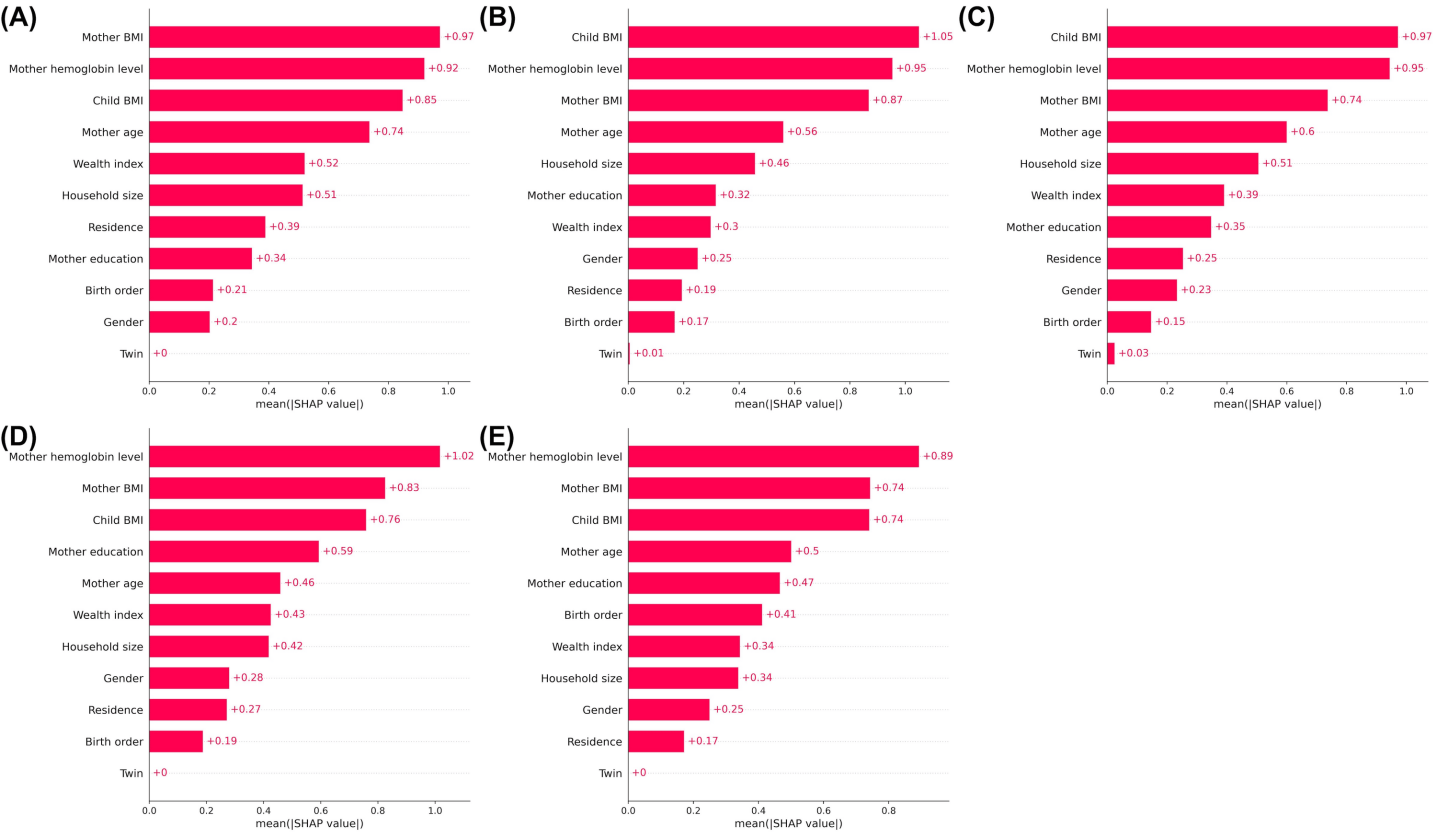
The ranking of the variables as per their importance based on the mean (|SHAP value|), stratified by age group Age groups: (A) 6–11 months, (B) 12–23 months, (C) 24–35 months, (D) 36–47 months, and (E) 48–59 months.

Notably, the maternal hemoglobin level was a significant predictor in the age-specific analysis (Figure 4).

Furthermore, maternal BMI and child's BMI may be important predictors for children younger than three years old.

## Discussion

The current study revealed a strong association between maternal anemia and anemia in the child. Additionally, children of mothers with anemia were approximately 1.5 to two times more likely to suffer from anemia compared to those of mothers with no anemia. Maternal anemia appears as a significant predictor for anemia in the child, after adjusting for other various covariates.

Maternal anemia was determined as a key predictor, following the child's age, in the SHAP framework. Previous research conducted in Myanmar [16] and Zimbabwe [17] revealed that maternal anemia may be a crucial predictor of anemia in the child [16]. Several risk factors, including the child's age, the child being underweight, being born as a twin, and maternal anemia, education level, and household size, were identified as being associated with anemia in the child, from studies based on Nepal DHS [9-11]. However, the relative importance of maternal anemia compared with other predictors remained unclear. This study confirmed maternal anemia as a key predictor, even when considered alongside other variables. The primary importance of the child's age is biologically intuitive, as iron requirements are highest during the rapid growth phase of infancy, making younger children inherently more vulnerable [18]. Notably, maternal anemia emerged as a more powerful predictor than socioeconomic variables such as maternal education or household wealth. This suggests that while socioeconomic factors create the broader environment influencing the child's nutrition, the mother's own physiological iron status represents a more direct and immediate risk factor, likely through mechanisms like shared dietary patterns, genetic predispositions, and the iron content of breast milk.Further studies are warranted to evaluate the consistency of these results, based on the data from Nepal, across different geographical contexts.

The influence of maternal education and other socioeconomic factors on anemia prevalence is a critical aspect of the association between maternal anemia and anemia in the child. Maternal education is strongly linked to improved health literacy, enabling mothers to make better-informed decisions regarding nutrition and healthcare, for themselves and their children [19]. For instance, educated mothers may have a greater knowledge of iron-rich foods and infant feeding practices. Furthermore, household wealth, a key socioeconomic determinant, affects access to nutritious food, quality healthcare, and better sanitation, all of which are protective against anemia. Intra-household resource allocation, which can be influenced by both maternal education and wealth, also plays a role in determining the nutritional intake of a child. Our findings, which show maternal education and wealth index as significant predictors in the SHAP model, align with studies from other settings that highlight the critical role of socioeconomic determinants in childhood anemia [20].

Additionally, a plausible biological mechanism linking maternal anemia to anemia in the child is the potentially lower iron concentration in the breast milk of anemic mothers, which could lead to insufficient iron intake for the infant [21]. This hypothesis is supported by a prospective observational study from a teaching hospital in central India, which found that the iron concentration in the breast milk correlated with maternal hemoglobin and iron levels and was significantly reduced in severely anemic mothers [22]. However, the evidence is not conclusive, as other studies have reported no relationship between iron levels in the breast milk and the mother's hemoglobin or iron status [23]. Given these conflicting findings, the extent to which insufficient iron in the breast milk serves as a primary driver of childhood anemia is not yet fully elucidated and warrants further research.

Our study's findings can directly inform and potentially enhance the effectiveness of the existing programs. The Government of Nepal, through strategies like the "Nepal Health Sector Programme," has implemented several key interventions to combat anemia [24]. These include iron and folic acid supplementation for pregnant women, the distribution of micronutrient powders for children, biannual deworming, and nutritional counseling. Despite these efforts, the prevalence of moderate-to-severe childhood anemia remains a significant public health challenge, fluctuating between 19% and 27% in recent surveys [8]. The strong predictive link between maternal anemia and anemia in the child suggests that programs addressing these issues in isolation may have limited success. Specifically, a mother's anemia diagnosis during antenatal or postnatal care should serve as a critical flag, automatically triggering screening and prioritized nutritional support for her child. Our analysis provides evidence to support a strategic shift toward a more integrated, dyadic approach. This targeted strategy would allow for more efficient allocation of resources, such as micronutrient powders and counseling, to the most at-risk households. By viewing and tackling maternal anemia and anemia in the child as an interconnected problem, the impact of Nepal's national health programs could be significantly strengthened.

In this study, the prevalence of anemia was higher in the younger age groups, with 40% of children under one year of age having moderate-to-severe anemia. This trend was consistent with surveys from other countries [11], indicating that the major factors contributing to the development of anemia may vary by age group. Therefore, implementing age-specific measures may be necessary. Conversely, this study also revealed that maternal anemia was an important predictor of anemia in children, regardless of age.

The SHAP framework is a valuable methodology in the presence of several predictors, and the importance of each determinant needs to be evaluated [13,25]. The SHAP methodology was used to compute Shapley values, which is a concept borrowed from the cooperative game theory approach, to quantify the contribution of each variable to the model’s prediction. This is accomplished by evaluating the model across multiple combinations of variables and computing the average difference in the model’s output when including versus excluding a particular variable. This computed difference, also known as the Shapley value, represents the average marginal contribution of a feature to the model’s prediction. Consequently, the collection of Shapley values provides a measurable indication of each variable’s contribution to the model’s prediction for a specific input, facilitating a better understanding of the importance of specific variables.

Finally, while our study is focused on Nepal, its findings likely have broader relevance for other LMICs where anemia remains a persistent public health challenge. The strong predictive link between maternal anemia and anemia in the child is not unique to Nepal; similar associations have been documented in diverse settings across South Asia, such as India [21,22], Bangladesh [20], and sub-Saharan Africa [17]. These regions often share key underlying risk factors with Nepal, including widespread iron-deficient diets, high burdens of infectious disease, and limited access to healthcare. Therefore, the core principle of our recommendation, using maternal anemia as a practical, high-precision trigger for child screening and intervention, is a potentially transferable and scalable strategy. Health systems in other LMICs could adapt this dyadic approach to their own contexts, leveraging existing community health worker programs, similar to Nepal's Female Community Health Volunteers, to improve the efficiency and impact of their anemia control efforts. Future multi-country studies would be valuable to formally validate the generalizability of our model's predictive weights and to tailor this intervention strategy to different cultural and health system contexts.

Our study has several limitations. First, the SHAP value is dependent on the current prediction model, and the importance of the predictors may vary in different prediction frameworks. Future studies are warranted to evaluate the importance of maternal anemia as a predictive factor in a variety of alternative models. Additionally, SHAP values should be interpreted with caution, as they indicate only predictive importance, not causality. Because this study is also cross-sectional in design, causal claims are strictly prohibited. Second, this survey did not identify the cause of anemia among both the mother and child. The association between anemia in the mother and her child may vary depending on the etiology of the anemia. Third, our analysis was constrained by the variables available in the DHS datasets. Consequently, we were unable to explore the potential impact of other important factors known to influence anemia, such as detailed maternal dietary patterns (e.g., iron intake), subclinical infections, or specific environmental exposures. These unmeasured variables represent an important area for future primary research. Fourth, our definition of anemia (hemoglobin <10.0 g/dL) was intentionally stricter than the standard WHO threshold (<11 g/dL) in order to focus our analysis on moderate-to-severe cases, which are of greater clinical significance. However, this approach has two important consequences. It limits the direct comparability of our prevalence estimates with those from numerous studies that adhere to the WHO standard, and it likely results in an underestimation of the total burden of childhood anemia, as cases of mild anemia were excluded. Finally, blood samples collected by capillary puncture were used to measure hemoglobin levels, which may cause measurement errors due to testing techniques. Capillary sampling can introduce variability and may yield hemoglobin values that differ from venous samples, potentially leading to misclassification of anemia status [26], particularly for children with values near the 10 g/dL cutoff.

## Conclusions

This study confirms that maternal anemia is a powerful predictor of anemia in the child in Nepal. Reinforcing this finding, our SHAP analysis identified maternal anemia as the second most influential predictor, surpassed only by the child's age, thereby highlighting its critical role among a multitude of risk factors.

These findings have direct implications for both clinical practice and public health strategy. Clinically, a mother's diagnosis of anemia should be considered a critical indicator that triggers immediate screening of her child. Consequently, public health interventions must evolve beyond a singular focus on the child. Effective strategies to reduce the burden of childhood anemia will require comprehensive, household-level approaches that target the nutritional health of both mothers and their children. Such interventions could include integrating anemia screening for the child into routine postnatal care for mothers diagnosed with anemia, providing iron supplementation to both the mother and child as a unit, and delivering household-based nutritional education. Implementing these strategies in a context like Nepal requires overcoming logistical challenges and ensuring they are culturally appropriate. Addressing maternal anemia is therefore an essential and foundational step toward improving child health outcomes in Nepal and likely in other regions with a high burden of anemia.
